# Upper airway viruses and bacteria in urban Aboriginal and Torres Strait Islander children in Brisbane, Australia: a cross-sectional study

**DOI:** 10.1186/s12879-017-2349-1

**Published:** 2017-04-04

**Authors:** Kerry-Ann F. O’Grady, Kerry K. Hall, Theo P. Sloots, Jennie Anderson, Anne B. Chang

**Affiliations:** 1 Institute of Health & Biomedical Innovation, Centre for Children’s Health Research, Queensland University of Technology, 62 Graham Street, South Brisbane, QLD 4101 Australia; 2 Child Health Research Centre, Centre for Children’s Health Research, The University of Queensland, 62 Graham Street, South Brisbane, QLD 4101 Australia; 3 Caboolture Community Medical, King Street, Caboolture, QLD 4501 Australia; 4 Menzies School of Health Research, Charles Darwin University, Rocklands Drive, Tiwi, Northern Territory, 0810 Australia; 5 Department of Respiratory Medicine, Lady Cilento Children’s Hospital, Stanley Street, South Brisbane, QLD 4101 Australia

**Keywords:** Aboriginal and Torres Strait Islander, Child, Respiratory, Nasal carriage, Viruses, Bacteria, Prevalence

## Abstract

**Background:**

Respiratory morbidity in Australian Indigenous children is higher than their non-Indigenous counterparts, irrespective of urban or remote residence. There are limited studies addressing acute respiratory illness (ARI) in urban Indigenous children, particularly those that address the upper airway microbiome and its relationship to disease. We aimed to describe the prevalence of upper airway viruses and bacteria in symptomatic and asymptomatic urban-based Australian Indigenous children aged less than 5 years.

**Methods:**

A cross-sectional analysis of data collected at baseline in an ongoing prospective cohort study of urban Aboriginal and Torres Strait Islander children registered with a primary health care service in the northern suburbs of Brisbane, Australia. Clinical, demographic and epidemiological data and bilateral anterior nasal swabs were collected on enrolment. Polymerase chain reaction was performed on nasal swabs to detect 17 respiratory viruses and 7 bacteria. The primary outcome was the prevalence of these microbes at enrolment. Logistic regression was performed to investigate differences in microbe prevalence between children with and without acute respiratory illness with cough as a symptom (ARIwC) at time of specimen collection.

**Results:**

Between February 2013 and October 2015, 164 children were enrolled. The median age at enrolment was 18.0 months (IQR 7.2–34.3), 49.4% were boys and 56 children (34.2%) had ARIwC. Overall, 133/164 (81%) nasal swabs were positive for at least one organism; 131 (79.9%) for any bacteria, 59 (36.2%) for any virus and 57 (34.8%) for both viruses and bacteria. Co-detection of viruses and bacteria was more common in females than males (61.4% vs 38.6%, *p* = 0.044). No microbes, alone or in combination, were significantly associated with the presence of ARIwC.

**Conclusions:**

The prevalence of upper airways microbes in asymptomatic children is similar to non-Indigenous children with ARIwC from the same region. Determining the aetiology of ARIwC in this community is complicated by the high prevalence of multiple respiratory pathogens in the upper airways.

**Study registration:**

Australia New Zealand Clinical Trial Registry Registration Number: 12,614,001,214,628. Retrospectively registered.

**Electronic supplementary material:**

The online version of this article (doi:10.1186/s12879-017-2349-1) contains supplementary material, which is available to authorized users.

## Background

Acute and chronic respiratory illnesses are predominant causes of morbidity and mortality in Aboriginal and Torres Strait Islander (hereforth respectfully called Indigenous) children in Australia [1]. In some remote regions of Australia, Indigenous infants present on average at least once a fortnight to community clinics and acute respiratory illnesses (ARI) are the most common reason for attendance [2]. Indigenous children are 2.6 times more likely to present to emergency departments (ED) with ARI [3] and twice as likely to be hospitalised for an ARI than non-Indigenous children [1]. However, respiratory research in Indigenous children has predominantly focused on remote-based children [4, 5], although the majority of Indigenous children live in urban or inner regional areas of Australia, with Brisbane having the largest of those communities [6]. There is a conspicuous lack of current data on ARI and related respiratory microbes in urban Indigenous children at the community level and it is unknown whether it is similar to remote children or non-Indigenous children in urban settings. Further, cough is the most common symptom associated with health care utilisation amongst Australian children [7], including Indigenous children and, if present in ARI, is likely indicative of a lower ARI particularly if wet.

Establishing the microbiological aetiology of ARI with cough (ARIwC) in children is complex, particularly if upper airway specimens are used [8], given many organisms are also detected in the nasopharynx of healthy children. However upper airway microbial data obtained from the nasopharynx are still considered important because they can provide important epidemiological information on the prevalence of, and temporal trends in, organisms within and between different populations. Knowing upper airway microbial epidemiology is particularly important to informing public health strategies such as vaccination. Increasingly co-detection of viruses with bacteria is considered important and a recent South African study reported 5 different types of organisms per episode were found in the upper airways of young children [9]. Yet, there are limited data on factors that are associated with virus and bacteria co-detection in Indigenous children, particularly those in urban settings.

Thus, in 164 urban-based Indigenous children presenting to an urban primary health care service, we described the prevalence of upper airway respiratory viruses and bacteria. We also sought to identify factors associated with virus-bacteria codetection. We hypothesised that virus-bacteria codetection was more likely in children with ARIwC than those without.

## Methods

### Design and setting

We analysed data from a cohort of urban Aboriginal and Torres Strait Islander children aged less than 5 years collected at time of enrolment into a prospective study of ARIwC. The full protocol of the prospective study has been previously published [10]. The study was conducted in a large primary healthcare clinic in the northern suburbs of subtropical Brisbane which has a patient population of approximately 11,500 people. Fifty-nine percent of the patients identify as being Indigenous.

### Recruitment and data collection

An Aboriginal research officer approached all children aged less than five years and their parent or guardian at time of presentation to the clinic for any reason (including well child checks and accompanying another person presenting for health care). Children were eligible for inclusion in the primary cohort study [10] if they were: a) identified by the parent/guardian as being Indigenous; b) a regular patient of the clinic; c) aged less than five years at time of enrolment, and; d) parents/guardians were willing and able to complete the study requirements. The reason for presentation and the presence of ARIwC were not determined until after the child had been enrolled. For the analysis presented in this manuscript, only children who had a nasal swab performed were included. There were no exclusion criteria.

At enrolment, detailed demographic, environmental, clinical and socio-economic data were collected, and an anterior bilateral nasal swab was performed. Nasal swabs were collected using the Virocult**™** system (Medical Wire and Equipment, Corsham, UK) by inserting the tip at least 1 cm into each nare and turning the swab four times against the nasal mucosa. A child was considered symptomatic of ARIwC if any of the following symptoms occurred within seven days prior to and including the day of enrolment: cough **and** other local or systemic symptoms suggestive of a respiratory illness (eg. runny nose, wheeze, dyspnoea and tachypnoea). Children did not meet the case definition if cough was not present during that time.

### Laboratory methods

Nasal swabs were stored refrigerated until they were transferred within one week to -80 °C freezers. Multiplex polymerase chain reaction (PCR) was used to test for adenovirus, respiratory syncytial virus (RSV) groups A and B, influenza virus types A and B, parainfluenza virus types 1–3, human metapneumovirus, human rhinoviruses, human coronaviruses (OC43, 229E,NL63 + HKU1), human bocavirus, human polyomaviruses KI and WU, *M. pneumoniae, C. pneumoniae, B. pertussis, S. pneumoniae*, *S. aureus,* non-typeable *Haemophilus influenzae* (NTHi) and *M. catarrhalis* using previous established methods [11, 12].

### Data analyses

Descriptive analyses were performed with data expressed as proportions and/or means of the selected characteristics. Where continuous data were not normally distributed, medians with accompanying interquartile ranges are presented. Univariate analyses were performed to evaluate potential differences in child characteristics between swabs in which codetection of virus and bacteria did and did not occur. Chi_2_ statistics were used to assess differences in proportions and the Wilcoxon Rank Sum test was used to compare differences in medians. Given the lack of sufficient significant findings on univariate analyses, regression analyses were not performed. All analyses were performed in Stata V14SE (StataCorp, College Station, TX, USA).

## Results

Between February 2013 and November 2015, 403 children were screened and 180 Indigenous children were enrolled. Reasons for non-enrolment were 20 (4.9%) were non-Indigenous, 72 (17.9%) declined, 43 (10.7%) were ineligible and 88 (21.8%) were not enrolled for other reasons. There were no differences in age and gender between children who were and were not enrolled. One child was withdrawn as a screen failure, 15 children/parents refused specimen collection and thus nasal swabs were collected from 164 children. Of the 164 children, the median age at enrolment was 18.0 months (interquartile range (IQR) 7.2–34.3) and 49.4% were male; respiratory symptoms at time of enrolment were present in 56 children (34.2%). There were no differences in the median ages of children with and without ARIwC (*p* = 0.432) nor any differences within and between age groupings (ie. < 6 months, 6 - <12 months, 12 - <24 months and 24+ months; *p* = 0.529).

Overall 133/164 (81%) nasal swabs were positive for at least one organism, 131 (79.9%) for any bacteria, 59 (36.2%) for any virus and 57 (34.8%) for both viruses and bacteria (Table 1). All but two swabs that were positive for one or more viruses also had bacteria detected. Three or more organisms were detected in 33.1% of swabs. *C. pneumoniae* and *M. pneumoniae* were not detected in any specimens and are not considered further. There were no statistical differences in the prevalences of any virus or bacterium, alone or in combination, between children with and without ARIwC (Table 1). Even in children without ARIwC, a virus was detected in 36.5% of children.

**Table 1:** Prevalence of viruses and bacteria detected in the nasal swabs of 164 urban Indigenous children aged less than 5 years by presence of ARI with cough

	All episodes	Symptomatic (56)	Asymptomatic (108)	*p* value
	n (%)	n (%)	n (%)	
Any organism positive	132 (81.1)	44 (78.6)	88 (81.5)	0.522
Total organisms positive				
0	31 (19.0)	12 (21.4)	19 (17.8)	0.493
1	40 (24.4)	15 (26.8)	25 (23.1)	
2	37 (22.7)	15 (26.8)	22 (20.6)	
3	25 (15.3)	6 (10.7)	19 (17.8)	
4	20 (12.3)	4 (7.1)	16 (15.0)	
5	9 (5.5)	4 (7.1)	5 (4.7)	
NTHi + any other organism	40 (23.4)	12 (21.4)	28 (25.9)	0.525
NTHi only	13 (7.9)	4 (7.1)	9 (8.3)	0.789
NTHi + Mcat only	4 (2.4)	1 (1.8)	3 (2.8)	1.000
NTHi + Spn only	4 (2.4)	2 (3.6)	2 (1.9)	0.606
NTHi + Mcat + Spn only	5 (3.1)	1 (1.8)	4 (3.7)	0.662
NTHi only + Any virus	25 (15.2)	8 (14.3)	17 (15.7)	0.806
NTHi + Mcat + Any virus	19 (11.6)	6 (10.7)	13 (12.0)	0.802
NTHi + Spn + Any virus	20 (12.2)	6 (10.7)	14 (12.7)	0.676
NTHi + Mcat + Spn + Any virus	15 (9.2)	4 (7.1)	11 (10.2)	0.522
Spn + any other organism	88 (53.7)	29 (51.8)	59 (54.6)	0.729
Spn only	4 (2.4)	2 (3.6)	2 (1.9)	0.606
Spn + Mcat only	9 (5.5)	2 (3.6)	7 (6.5)	0.720
Spn only + any virus	2 (1.2)	2 (3.6)	0	-
Spn + Mcat + Any virus	8 (4.9)	2 (3.6)	6 (5.6)	0.717
Mcat + any other organism	78 (47.6)	26 (46.4)	52 (48.2)	0.834
Mcat only	4 (2.4)	2 (3.6)	2 (1.9)	0.606
Mcat only + Any virus	8 (4.9)	2 (3.6)	6 (5.6)	0.717
Saur	32 (19.5)	7 (12.5)	25 (23.2)	0.103
Saur only	10 (6.1)	0	10 (9.3)	-
Bpert + any other	1 (0.6)	0	1 (0.9)	-
Bpert only	0	0	0	-
Any bacteria positive (not excl virus)	131 (79.9)	42 (75.0)	89 (82.4)	0.262
Any bacteria positive (excl virus)	81 (49.4)	27 (48.2)	54 (50.0)	0.828
Number bacteria positive (not excl virus)				
0	33 (20.1)	14 (25.0)	19 (17.6)	0.657
1	53 (32.3)	18 (32.1)	35 (32.4)	
2	49 (29.9)	17 (30.4)	32 (29.6)	
3	26 (15.9)	6 (10.7)	20 (18.5)	
4	3 (1.8)	1 (1.8)	2 (1.9)	
RSV + any organism	6 (3.7)	2 (3.6)	4 (3.7)	0.957
RSV only	0	0	0	
Human rhinovirus + any organism	29 (17.8)	9 (16.1)	20 (18.7)	0.678
Human rhinovirus only	0	0	0	
Adenovirus + any other organism	5 (3.1)	2 (3.6)	3 (2.8)	0.787
Adenovirus only	0	0	0	
Human metapneumovirus + any other	2 (1.2)	1 (1.8)	1 (0.9)	1.000
Human metapneumovirus only	0	0	0	
Parainfluenzae types 1–3 + any other	6 (3.7)	2 (3.6)	4 (3.7)	1.000
Parainfluenzae types 1–3 only	0	0	0	
Influenza + any organism	1 (0.6)	1 (1.8)	0	-
Influenza only	0	0	0	
Bocavirus + any organism	11 (6.8)	3 (5.4)	8 (7.5)	0.608
Bocaonly	0	0	0	
Human coronaviruses + any organism	5 (3.1)	2 (3.6)	3 (2.8)	1.000
Human coronaviruses only	0	0	0	
Polyomaviruses + any organism	8 (4.9)	3 (5.4)	5 (4.7)	1.000
Polyomaviruses only	1 (2.3)	1 (1.8)	0	-
Any virus positive (not excl bacteria)	59 (36.2)	20 (35.7)	39 (36.5)	0.926
Any virus positive (no bacteria)	2 (2.3)	2 (3.6)	0 (0)	-
Number virus positive (+ any bacteria)				
0	104 (63.8)	36 (64.3)	68 (63.6)	0.959
1	47 (28.8)	16 (28.6)	31 (29.0)	
2	10 (6.1)	3 (5.4)	7 (6.5)	
3	2 (1.2)	1 (1.8)	1 (0.9)	
Virus and bacteria positive	57 (34.8)	18 (32.1)	39 (36.1)	0.613

*NTHI* non-typeable *H. influenza*e, *Spn S. pneumoniae, Mcat M. catarrhalis, Saur S. aureus*, *Bpert B. pertussis, RSV* respiratory syncytial virus

Virus-bacteria co-detection was more frequent in females than males (61.4% vs 38.6%, *p* = 0.044) and this was not age-dependent. No other characteristics were significantly different between children with and without codetection of viruses and bacteria (Table 2). The prevalences of specific bacterium – bacterium, virus – virus and virus-bacterium codetections are presented in the Additional file 1: Table S1.

**Table 2:** Child characteristics by codetection of both viruses and bacteria in nasal swabs from 164 urban Indigenous children aged less than 5 years

	All episodes	Codetection	No codetection	*p*-value
	(*N* = 164)	(*N* = 57)	(*N* = 107)	
	n (%)	n (%)	n (%)	
Gender				
Male	81 (49.4)	22 (38.6)	59 (55.1)	0.044
Female	83 (50.6)	35 (61.4)	48 (44.9)	
Median age in months (IQR)	18.0 (7.2–34.3)	17.7 (10.4–25.8)	18.2 (6.3–39.1)	0.511
Agegroup (Months)				
< 6	32 (19.5)	7 (12.3)	25 (23.4)	0.529
6 - <12	26 (15.9)	9 (15.8)	17 (15.9)	
12 - <24	43 (26.2)	23 (40.3)	20 (18.7)	
24 - <60	63 (38.4)	18 (31.6)	45 (42.1)	
Total annual household income (AUD)				
> =$78,000	16 (9.8)	5 (8.8)	11 (10.3)	0.947
$52,000 - < $78,000	25 (15.2)	10 (17.5)	15 (14.0)	
$26,000 - < $52,000	62 (37.8)	21 (36.8)	41 (38.3)	
< $26,000	61 (37.2)	21 (36.8)	40 (37.4)	
Gestational age				
> = 37 weeks	145 (88.4)	9 (15.8)	10 (9.4)	0.220
< 37 weeks	19 (11.6)	48 (84.2)	97 (90.7)	
Birth weight				
> =2500 g	135 (82.3)	45 (78.9)	90 (84.1)	0.409
< 2500 g	29 (17.7)	12 (21.1)	17 (15.9)	
Breastfeeding history				
Ever breastfed	119 (72.6)	41 (71.9)	78 (72.9)	0.895
Never breastfed	45 (27.4)	16 (28.1)	29 (27.1)	
Child care attendance				
Yes	49 (29.9)	21 (36.8)	28 (26.2)	0.155
No	115 (70.1)	36 (63.2)	79 (73.8)	
Number of other children in the house				
0	33 (20.1)	14 (24.6)	19 (17.8)	0.239
1–2	93 (56.7)	32 (56.1)	61 (57.0)	
> =3	38 (23.2)	11 (19.3)	27 (25.2)	
Pets in household				
Yes	94 (57.3)	35 (61.4)	59 (55.1)	0.440
No	70 (42.7)	22 (38.6)	48 (44.9)	
Regular exposure to environmental tobacco smoke				
Yes	119 (72.6)	44 (77.2)	75 (70.1)	0.332
No	45 (27.4)	13 (22.8)	32 (29.9)	
Respiratory symptom at time of swab				
Yes	56 (34.1)	18 (31.6)	38 (35.5)	0.613
No	108 (65.9)	39 (68.4)	69 (64.5)	
Season of enrolment				
Summer	38 (23.2)	12 (21.0)	26 (24.3)	0.500
Autumn	50 (30.5)	15 (26.3)	35 (32.7)	
Winter	35 (21.3)	16 (28.1)	19 (17.8)	
Spring	41 (25.0)	14 (24.6)	27 (25.2)	

**Fig. 1:**
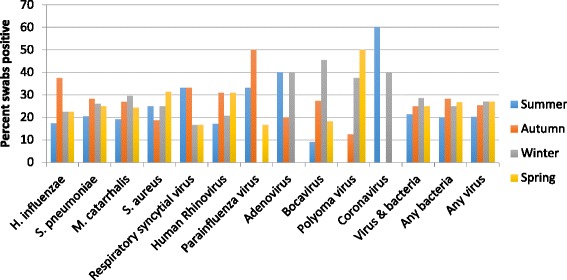
Seasonal distribution of upper airway viruses and bacteria in a cohort of urban Aboriginal and Torres Strait Islander children (*n* = 164)

The seasonal distribution of organisms detected is presented in Fig. 1; influenza and *B. pertussis* are not included given each was only detected in one swab over the entire study. All other bacteria were detected across all seasons of the year however this did not occur for the parainfluenza viruses, adenovirus, polyomaviruses and coronaviruses.

## Discussion

Given the lack of microbiological data in the upper airways of urban-based Indigenous children, we investigated this in a cohort of children aged <5 years attending a primary health care service with and without ARIwC. Irrespective of the reason for presentation to the clinic, a third of the children had ARIwC symptoms at enrolment. The prevalence of upper airway respiratory viruses and bacteria were very high; at least one virus or bacteria was detected in 81% of children and ≥3 organisms were detected in a third. The detection of any upper airway viruses and/or bacteria, alone or in combination, was similar between children with and without ARIwC and was not associated with age.

The prevalence of any organism in children in this study of 81% is similar to children aged <15 years presenting acutely to a tertiary paediatric emergency department (ED) in the same geographical location with ARIwC (91%), the majority of whom were non-Indigenous [13]. The prevalence of co-detection of viruses and bacteria in the ED study was 51.8% [13], higher than the 34.8% in this cohort. However, children in the ED study were all symptomatic for ARIwC whilst the majority of children in this current study were asymptomatic. The two studies utilised the same specimen collection and laboratory methods and tested for the same organisms at the research laboratory. In both studies, *S. pneumoniae* followed by *M.catarrhalis* and NTHi were the dominant bacteria and rhinoviruses were the most common virus. *B. pertussis, M. pneumoniae and C. pneumoniae* were rare and influenza was uncommon. Respiratory syncytial virus was only observed in 4 % of children in this study but was detected in 17% of children in the ED study [14]. In that study, RSV was weakly associated with children being hospitalised [14] and its higher prevalence amongst ED children compared to community children possibly reflects the severity of illness if infected rather than community prevalence. The seasonal distribution of organisms was also similar to that identified in the ED study [13], including a predominance of the autumn months for NTHi and RSV and that virus-bacteria codetection occurred predominantly in autumn and winter.

In a study of upper airway viruses and bacteria in Central Australian Aboriginal children hospitalised for pneumonia [8], a population with high rates of hospitalised lower ARI [15] and nasal colonisation [16], the overall prevalence of any organism was 94.5%, with 34.5% positive for both viruses and bacteria. The prevalences of *S. pneumoniae*, *M. catarrhalis* and NTHi were 64%, 70.3% and 76.5% respectively [8]. That study differed from the two Brisbane studies in that the NT study focused on children hospitalised with pneumonia rather than non-severe ARIwC, different specimen collection techniques were used, the PCR for bacteria was performed at a different laboratory and the Central Australian study was undertaken prior to widespread implementation of pneumococcal conjugate vaccines. However, more recent community based studies of nasopharyngeal carriage of these bacteria in the Northern Territory in the 13-valent pneumococcal conjugate vaccine era identified prevalences of 77% for *S. pneumoniae*, 45% for *M. catarrhalis* and 63% for NTHi [16]. Viruses were not reported in that study. In a Western Australian study of asymptomatic rural Aboriginal children that included testing for the same viruses as our study with the exception of bocavirus and the polyomaviruses [17], viruses were detected in 41% of children (most commonly rhinoviruses: 23.6%). Thus our data suggest that viral infection may be comparable between urban and remote Indigenous children however bacterial carriage is likely to be higher in remote children.

We identified only two swabs that were positive for viruses only (i.e. most had virus with bacteria co-detection); one was a single isolation of a polyomavirus and the other was a co-detection of rhinovirus and bocavirus. The reasons why we found so few virus-only detections are uncertain, particularly given few data in the literature that have tested for the same spectrum of organisms by PCR that was undertaken in this study. Both swabs were from children with ARIwC at the time of testing but the clinical significance of virus only detections is unknown. A recent study of respiratory viruses (*n* = 15) detected by PCR in 560 paediatric episodes of ARI reported 457 episodes were virus positive, of which 331 were single infections and 126 were multiple infections; testing was undertaken for only two bacteria (*C. pneumoniae* and *M. pneumoniae*) [18]. There was no difference in clinical severity and management between children with single infections and those with multiple infections.

We found no relationship between the child characteristics and virus-bacteria codetection other than gender. Notably there were no differences in codetection between children with and without ARIwC, although the lack of difference may be attributable to a secondary analysis of data and hence lack of power to identify important differences. However, there are limited studies that have evaluated a similar spectrum of organisms by PCR methods that included children with and without ARIwC at the community level. A Finnish study of 426 children aged 6 to 35 months with and without acute otitis media reported a number of associations between several viruses and fever, nasal congestion, rhinitis and cough [19]. *M. catarrhalis* in the presence of viruses was also associated with rhinitis, nasal congestion and cough [19]. In 161 Norwegian children attending two daycare centres over a 2-year period, nasopharyngeal swabs (NPS) were collected over 4 time points and analysed by PCR for *B. pertussis, M. pneumoniae, C. pneumoniae* and 16 viruses [20]. Overall 43% of 343 specimens were positive for at least one virus and none were positive for the three bacteria. In 331 swabs collected from 355 children who underwent a clinical examination, 70% of children with clear signs of respiratory tract infection were virus positive, compared to 41% with mild findings and 30% in those who were asymptomatic (*p* < 0.001), with rhinovirus the most common virus detected in all 3 groups [20]. In a study of respiratory viruses in Alaska Native children hospitalised with acute lower respiratory infections and age-matched community controls, viruses were detected by PCR in NPS in 90% of 440 hospitalized children and 52% of 425 asymptomatic community controls, with rhinoviruses the most common in both groups [21]. Bacteria were not reported in that study. While comparisons between children across studies are problematic given differences in demographics, geography and study methods, our study and those above emphasise the complexity in assigning ARIwC causality based on nasal specimens in children given the high prevalence of multiple organisms in asymptomatic children. The probable exceptions are RSV, influenza virus and human metapneumovirus given their relatively strong association with severe ARI in children and a low prevalence in asymptomatic children in several studies [22]. As these viruses were uncommon in our study it was not possible to examine their role in symptomatic respiratory infections.

Our study has limitations given the cross-sectional nature of the analyses, the relatively small number of children enrolled and that given this was single centre study, the children who were enrolled may differ to the general population of urban Indigenous children in Australia posing a risk of selection bias. Our study children differed from national Indigenous statistics with respect to the high prevalence of exposure to environmental tobacco smoke and other household characteristics such as the high number of single parent households, low total annual household income and low levels of attendance at childcare [23]. Further, PCR detection of viruses and bacteria does not necessarily equate to active infection at the time of testing and simply provides an indication of recent exposure to the organism. Next generation sequencing holds promise for the improved detection and differentiation of respiratory pathogens [24]. however the tests are costly which currently limits the use of the technology in population-based studies.

## Conclusions

Our study is the first to report upper airway microbial in urban-based Indigenous children with and without ARIwC that includes the range of microbes we tested for. With the exception of RSV, the prevalence of upper airway respiratory viruses and bacteria in urban Indigenous children is comparable to acutely unwell non-Indigenous children from the same urban area but differs from remote Indigenous children with respect to the latter having a higher prevalence of respiratory bacteria. Given the high prevalence (82%) of organisms detected in children without ARI, upper airway microbiology in urban-based Indigenous children should be interpreted with caution.

## Additional file

Additional file 1: Table S1. Codetection of upper airway viruses and bacteria in 164 urban Aboriginal and Torres Strait Islander children (DOCX 93 kb).

## Abbreviations

ARI: Acute respiratory illness
CI: Confidence interval
ED: Emergency department
IQR: Interquartile range
NPS: Nasopharyngeal swab
NTHi: Non-typeable *Haemophilus influenzae*
PCR: Polymerase chain reaction
RSV: Respiratory syncytial virus
UK: United Kingdom

## References

[1] Australian Institute of Health & Welfare (2015). **Aboriginal and Torres Strait Islander health performance framework 2014 report: detailed analyses.** Cat. No. IHW 167.

[2] Clucas DB, Carville KS, Connors C, Currie BJ, Carapetis JR, Andrews RM (2008). Disease burden and health-care clinic attendances for young children in remote aboriginal communities of northern Australia. Bull World Health Organ.

[3] Moore HC, de Klerk N, Jacoby P, Richmond P, Lehmann D (2012). Can linked emergency department data help assess the out-of-hospital burden of acute lower respiratory infections? **A population-based cohort study**. BMC Public Health.

[4] Moore H, Burgner D, Carville K, Jacoby P, Richmond P, Lehmann D (2007). Diverging trends for lower respiratory infections in non-aboriginal and aboriginal children. J Paediatr Child Health.

[5] O'Grady KAF, Chang AB (2010). Lower respiratory infections in Australian indigenous children. J Paediatr Child H.

[6] Australian Bureau of Statistics (2013). Estimates of aboriginal and Torres Strait Islander Australians, June 2011.

[7] Britt H, Miller GC, Henderson J, Bayram C, Harrison C, Valenti L, Wong C, Gordon J, Pollack AJ, Pan Y, et al. General practice activity in Australia 2014–2015. In: General practice series number 38. Sydney: The University of Sydney. p. 2015.

[8] Chang AB, Smith-Vaughan H, Sloots TP, Valery PC, Whiley D, Beissbarth J, Torzillo PJ (2015). Upper airway viruses and bacteria detection in clinical pneumonia in a population with high nasal colonisation do not relate to clinical signs. Pneumonia.

[9] Zar HJ, Barnett W, Stadler A, Gardner-Lubbe S, Myer L, Nicol MP (2016). Aetiology of childhood pneumonia in a well vaccinated South African birth cohort: a nested case-control study of the Drakenstein child health study. Lancet Respir Med.

[10] Hall KK, Chang AB, Sloots TP, Anderson J, Kemp A, Hammill J, Otim M, O'Grady KA (2015). The respiratory health of urban indigenous children aged less than 5 years: study protocol for a prospective cohort study. BMC Pediatr.

[11] O'Grady KA, Torzillo PJ, Rockett RJ, Whiley DM, Nissen MD, Sloots TP, Lambert SB (2011). Successful application of a simple specimen transport method for the conduct of respiratory virus surveillance in remote indigenous communities in Australia. Tropical Med Int Health.

[12] O'Grady KA, Whiley DM, Torzillo PJ, Sloots TP, Lambert SB (2013). Mailed versus frozen transport of nasal swabs for surveillance of respiratory bacteria in remote indigenous communities in Australia. BMC Infect Dis.

[13] O'Grady KF, Grimwood K, Sloots TP, Whiley DM, Acworth JP, Phillips N, Goyal V, Chang AB (2016). Prevalence, codetection and seasonal distribution of upper airway viruses and bacteria in children with acute respiratory illnesses with cough as a symptom. Clin Microbiol Infect.

[14] O’Grady KF, Grimwood K, Sloots TP. Upper airway viruses and bacteria and clinical outcomes in children with cough. Pediatr Pulmonol. 2016. in press. 10.1002/ppul.23527 PMC7167704 27458795

[15] O’Grady KA, Torzillo PJ, Chang AB (2010). Hospitalisation of indigenous children in the northern Territory for lower respiratory illness in the first year of life. Med J Aust.

[16] Leach AJ, Wigger C, Beissbarth J, Woltring D, Andrews R, Chatfield MD, Smith-Vaughan H, Morris PS (2016). General health, otitis media, nasopharyngeal carriage and middle ear microbiology in northern Territory aboriginal children vaccinated during consecutive periods of 10-valent or 13-valent pneumococcal conjugate vaccines. Int J Pediatr Otorhinolaryngol.

[17] Moore HC, Jacoby P, Taylor A, Harnett G, Bowman J, Riley TV, Reuter K, Smith DW, Lehmann D (2010). The interaction between respiratory viruses and pathogenic bacteria in the upper respiratory tract of asymptomatic aboriginal and non-aboriginal children. Pediatr Infect Dis J.

[18] Wishaupt JO, van der Ploeg T, de Groot R, Versteegh FG, Hartwig NG (2017). Single- and multiple viral respiratory infections in children: disease and management cannot be related to a specific pathogen. BMC Infect Dis.

[19] Uitti JM, Tahtinen PA, Laine MK, Huovinen P, Ruuskanen O, Ruohola A (2015). Role of nasopharyngeal bacteria and respiratory viruses in acute symptoms of young children. Pediatr Infect Dis J.

[20] Moe N, Pedersen B, Nordbo SA, Skanke LH, Krokstad S, Smyrnaios A, Dollner H (2016). Respiratory virus detection and clinical diagnosis in children attending day care. PLoS ONE.

[21] Singleton RJ, Bulkow LR, Miernyk K, DeByle C, Pruitt L, Hummel KB, Bruden D, Englund JA, Anderson LJ, Lucher L (2010). Viral respiratory infections in hospitalized and community control children in Alaska. J Med Virol.

[22] Shi T, McLean K, Campbell H, Nair H (2015). Aetiological role of common respiratory viruses in acute lower respiratory infections in children under five years: a systematic review and meta-analysis. J Global Health.

[23] Australian Institute of Health & Welfare (2015). The health and welfare of Australia’s aboriginal and Torres Strait Islander peoples: 2015.

[24] Graf EH, Simmon KE, Tardif KD, Hymas W, Flygare S, Eilbeck K, Yandell M, Schlaberg R (2016). Unbiased detection of respiratory viruses by use of RNA sequencing-based Metagenomics: a systematic comparison to a commercial PCR panel. J Clin Microbiol.

