# Supplementary material for: Upper airway viruses and bacteria in urban Aboriginal and Torres Strait Islander children in Brisbane, Australia: a cross-sectional study
Source: BMC Infect Dis. 2017 Apr 4;17:245. doi: 10.1186/s12879-017-2349-1 (PMC5381068; doi:10.1186/s12879-017-2349-1)
Supplement: Additional file 1: Table S1. — Codetection of upper airway viruses and bacteria in 164 urban Aboriginal and Torres Strait Islander children (DOCX 93 kb). [file 12879_2017_2349_MOESM1_ESM.docx]

**Table 3: Codetection of upper airway viruses and bacteria in 164 urban Aboriginal and Torres Strait Islander children**

|  | **NTHi** | **Spn** | **Mcat** | **Saur** | **Bpert** | **HRV** | **RSV** | **Flu** | **Paraflu** | **HMpv** | **Polyoma** | **Corona** | **Boca** | **Entero** |
| --- | --- | --- | --- | --- | --- | --- | --- | --- | --- | --- | --- | --- | --- | --- |
|  | **n (%)** | **n (%)** | **n (%)** | **n (%)** |  | **n (%)** | **n (%)** | **n (%)** | **n (%)** | **n (%)** | **n (%)** | **n (%)** | **n (%)** | **n (%)** |
| ***NT H. influenzae* (N = 40)** |  | 30 (75.0) | 28 (70.0) | 5 (12.5) | 1 (2.5) | 12 (30.0) | 3 (7.5) | 0 (0.0) | 4 (10.0) | 2 (5.0) | 3 (7.5) | 3 (7.5) | 8 (20.0) | 2 (5.0) |
| ***S. pneumoniae* (N = 88)** | 30 (34.1) |  | 49 (55.7) | 15 (17.1) | 0 (0.0) | 20 (22.7) | 3 (3.4) | 1 (1.1) | 5 (5.7) | 1 (1.4) | 6 (6.8) | 5 (5.7) | 11 (11.4) | 6 (6.8) |
| ***M. catarrhalis* (N=78)** | 28 (35.9) | 49 (62.8) |  | 11 (14.1) | 1 (1.3) | 23 (29.5) | 4 (5.1) | 0 (0.0) | 6 (7.7) | 2 (2.6) | 5 (6.4) | 5 (6.4) | 7 (9.0) | 9 (11.5) |
| ***S. aureus (*N = 31)** | 5 (15.6) | 15 (46.9) | 11 (34.4) |  | 0 (0.0) | 3 (9.7) | 1 (3.2) | 0 (0.0) | 1 (3.2) | 0 (0.0) | 2 (6.5) | 0 (0.0) | 2 (4.6) | 0 (0.0) |
| ***B. pertussis* (N = 1)** | 1 (100.0) | 0 (0.0) | 1 (100.0) | 0 (0.0) |  | 1 (100.0) | 0 (0.0) | 0 (0.0) | 0 (0.0) | 0 (0.0) | 0 (0.0) | 0 (0.0) | 0 (0.0) | 0 (0.0) |
| **Human rhinovirus (N = 29)** | 12 (41.4) | 20 (69.0) | 23 (79.3) | 3 (10.3) | 1 (3.5) |  | 0 (0.0) | 0 (0.0) | 0 (0.0) | 1 (3.5) | 1 (3.5) | 0 (0.0) | 3 (10.3) | 2 (6.9) |
| **Respiratory syncytical virus ( N= 6)** | 3 (50.0) | 3 (50.0) | 4 (66.7) | 1 (16.7) | 0 (0.0) | 0 (0.0) |  | 0 (0.0) | 0 (0.0) | 0 (0.0) | 0 (0.0) | 0 (0.0) | 1 (16.7) | 0 (0.0) |
| **Influenza virus (N = 1)** | 0 (0.0) | 1 (100.0) | 0 (0.0) | 0 (0.0) | 0 (0.0) | 0 (0.0) | 0 (0.0) |  | 0 (0.0) | 0 (0.0) | 0 (0.0) | 0 (0.0) | 0 (0.0) | 0 (0.0) |
| **Parainfluenza virus (N = 6)** | 4 (66.7) | 5 (83.3) | 6 (100.0) | 1 (16.7) | 0 (0.0) | 0 (0.0) | 0 (0.0) | 0 (0.0) |  | 0 (0.0) | 0 (0.0) | 0 (0.0) | 1 (16.7) | 0 (0.0) |
| **Human metapneumovirus (N = 2)** | 2 (100.0) | 1 (50.0) | 2 (100.0) | 0 (0.0) | 0 (0.0) | 1 (50.0) | 0 (0.0) | 0 (0.0) | 0 (0.0) |  | 0 (0.0) | 0 (0.0) | 1 (50.0) | 0 (0.0) |
| **Polyomaviruses (N = 8)** | 3 (37.5) | 6 (75.0) | 5 (62.5) | 2 (25.0) | 0 (0.0) | 1 (12.5) | 0 (0.0) | 0 (0.0) | 0 (0.0) | 0 (0.0) |  | 1 (12.5) | 1 (12.5) | 0 (0.0) |
| **Coronaviruses (N = 5)** | 3 (60.0) | 5 (100.0) | 5 (100.0) | 0 (0.0) | 0 (0.0) | 0 (0.0) | 0 (0.0) | 0 (0.0) | 0 (0.0) | 0 (0.0) | 1 (20.0) |  | 1 (20.0) | 2 (40.0) |
| **Bocavirus (N = 11)** | 8 (72.7) | 10 (90.9) | 7 (63.6) | 2 (18.2) | 0 (0.0) | 3 (27.3) | 1 (9.1) | 0 (0.0) | 1 (9.1) | 1 (9.1) | 1 (9.1) | 1 (9.1) |  | 2 (18.2) |
| **Enterovirus (N = 9)** | 2 (22.2) | 6 (66.7) | 9 (100.0) | 0 (0.0) | 0 (0.0) | 2 (22.2) | 0 (0.0) | 0 (0.0) | 0 (0.0) | 0 (0.0) | 0 (0.0) | 2 (22.2) | 2 (22.2) |  |
